# Advances in Doxorubicin-based nano-drug delivery system in triple negative breast cancer

**DOI:** 10.3389/fbioe.2023.1271420

**Published:** 2023-11-17

**Authors:** Weiwei Zeng, Yuning Luo, Dali Gan, Yaofeng Zhang, Huan Deng, Guohui Liu

**Affiliations:** ^1^ Department of Pharmacy, Shenzhen Longgang Second People’s Hospital, Shenzhen, Guangdong, China; ^2^ Shenzhen Longhua Maternity and Child Healthcare Hospital, Shenzhen, Guangdong, China

**Keywords:** doxorubicin, nanocarriers, triple negative breast cancer, drug delivery, clinical

## Abstract

Triple negative breast cancer (TNBC) is one of the most aggressive breast cancer. Due to the unique cell phenotype, aggressiveness, metastatic potential and lack of receptors or targets, chemotherapy is the choice of treatment for TNBC. Doxorubicin (DOX), one of the representative agents of anthracycline chemotherapy, has better efficacy in patients with metastatic TNBC (mTNBC). DOX in anthracycline-based chemotherapy regimens have higher response rates. Nano-drug delivery systems possess unique targeting and ability of co-load, deliver and release chemotherapeutic drugs, active gene fragments and immune enhancing factors to effectively inhibit or kill tumor cells. Therefore, advances in nano-drug delivery systems for DOX therapy have attracted a considerable amount of attention from researchers. In this article, we have reviewed the progress of nano-drug delivery systems (e.g., Nanoparticles, Liposomes, Micelles, Nanogels, Dendrimers, Exosomes, etc.) applied to DOX in the treatment of TNBC. We also summarize the current progress of clinical trials of DOX combined with immune checkpoint inhibitors (ICIS) for the treatment of TNBC. The merits, demerits and future development of nanomedicine delivery systems in the treatment of TNBC are also envisioned, with the aim of providing a new class of safe and efficient thoughts for the treatment of TNBC.

## 1 Introduction

Triple negative breast cancer (TNBC) is a specific type of breast cancer that lacks estrogen receptor (ER), progesterone receptor (PR), and human epidermal growth factor receptor-2 (HER2), and accounts for approximately 15% of all breast cancer cases (Kudelova et al., 2022; Leon-Ferre and Goetz, 2023). Compared with other breast cancer subtypes, TNBC is characterized by a high degree of malignancy, invasiveness, high rate of early recurrence and visceral metastasis, and difficulty in treatment, making it an extremely aggressive class of breast cancer subtype (Bianchini et al., 2022; Chowdhury et al., 2021). In addition, due to the lack of relevant receptors on the surface of TNBC cells, hormone receptor-positive patients are often insensitive to endocrine therapy/targeted therapy, which treatment is greatly therapeutically limited (Voelker, 2020; Mo and Xu, 2021). To address this dilemma, researchers are also continuously investigating novel drugs to prolong the survival of TNBC patients, such as immune checkpoint inhibitors (PD1/PD-L1 inhibitors (Elmakaty et al., 2023; Zhang M et al., 2022), poly (adenosine diphosphate) ribose polymerase (PARP) inhibitors (Gong R et al., 2023), antibody-drug-coupled compounds (ADCs) (Choi et al., 2021; Epaillard et al., 2023), and epidermal growth factor receptor (EGFR) inhibitors, etc. (You et al., 2021; Zhang C et al., 2022). Currently, targeted drugs (Olaparib) (Ahn et al., 2023) and PD-L1 inhibitors (Pembrolizumab) (Ganguly and Gogia, 2021) have been approved for the treatment of triple positive breast cancer, but targeted therapy requires patients to carry a BRCA gene mutation, and immunotherapy requires patients to have a Combined Positive Score (CPS) score ≥10. Both are narrow in scope, available to only about 30 and 38 percent of patients, respectively. It is far from meeting the TNBC treatment demand (Nanda et al., 2016). Hence, the principal treatment method is still chemotherapy. DOX can inhibit the synthesis of RNA and DNA, killing effect on tumor cells of various growth cycles. At present, the anthracycline-based DOX chemotherapy regimen has a high response rate to TNBC, it has better efficacy in patients with metastatic TNBC (mTNBC), which is a commonly used chemotherapeutic agent for the treatment of TNBC in clinics (Chan et al., 2019; Mobus et al., 2021). At present, based on the preclinical basic research of DOX, it is often prone to drawbacks such as poor water solubility, excessive clearance *in vivo* and instability. In addition, due to its lack of targeting, this has a greater detrimental effect on the normal tissues around the tumor and the heart, which to some extent limits the use of the chemotherapeutic drug DOX in the clinic (Zhao et al., 2018; Makwana et al., 2021).

**FIGURE 1:**
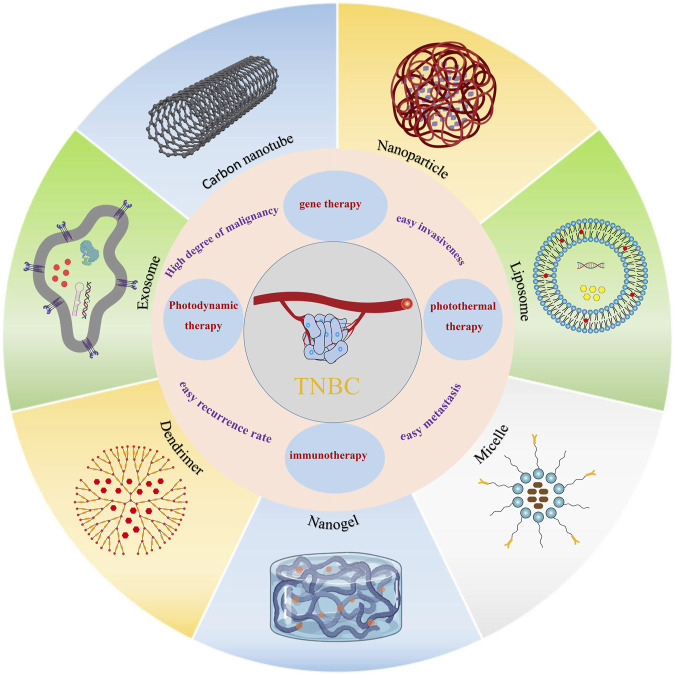
Schematic diagram of different nano-drug carriers for the release of DOX for the treatment of TNBC.

The development of nanomedicine has been driven by the urgent need for “efficient and low-toxicity” treatment of TNBC. Nano-drug delivery systems can not only achieve efficient loading and release of drugs, but also improve the enrichment rate and utilization of drugs in the lesion site. In addition, the controlled drug release can avoid the dispersion and sudden release of the drug in the treatment of TNBC to a certain extent. Furthermore, the nano-delivery system has the ability of nano-size, longer circulating half-life, higher drug encapsulation, surface modification ability, and targeting (active and passive) (Sudheesh et al., 2022; Mansour et al., 2023). Based on this, researchers have ingeniously leveraged the unique advantages of nano-delivery systems to work on novel formulations that have already made splash in the field of nanomedicine. Therefore, this paper reviews the novel nano-drug delivery systems (e.g., Nanoparticles, Liposomes, Micelles, Gels, Dendrimers, Exosomes, etc.) delivering chemotherapeutic drug DOX for the treatment of TNBC. We also summarize the progress of clinical trials of DOX in combination with immune checkpoint inhibitors (ICIS) for the treatment of TNBC. Meanwhile, we also discusses their strengths and weaknesses, and looks forward to their future directions of development, so that readers can have a more in-depth understanding of the progress of the research on DOX nanodelivery systems in TNBC. It is also hoped to provide new thoughts for the development of clinical application formulations of DOX, and to make a living for TNBC patients (Figure 1).

## 2 Novel nano-drug delivery systems

### 2.1 Nanoparticles

Nanoparticles are microscopic particles with a size of up to 100 nm. Nanoparticles have the characteristics of small size, excellent stability performance and biocompatibility as well as easy degradation in a short period of time, thus using nanoparticles as drug carriers for DOX, on the one hand, can improve the stability and solubility of the encapsulated DOX, promote transmembrane transport, and prolong the circulation time *in vivo* (Liu et al., 2022; Shokooh et al., 2022). On the other hand, the functionalization of nanoparticles can deliver DOX to the focal site of TNBC, slowing down the degradation of the drug, which improves the delivery efficiency and reduces the adverse effects to a certain extent (Liu et al., 2022; Chen et al., 2023; Kong et al., 2023). With the application of nanoparticle-based precision therapeutics in cancer medicine, researchers are striving to develop a DOX-nanoparticle delivery system for the treatment of TNBC.

**FIGURE 2:**
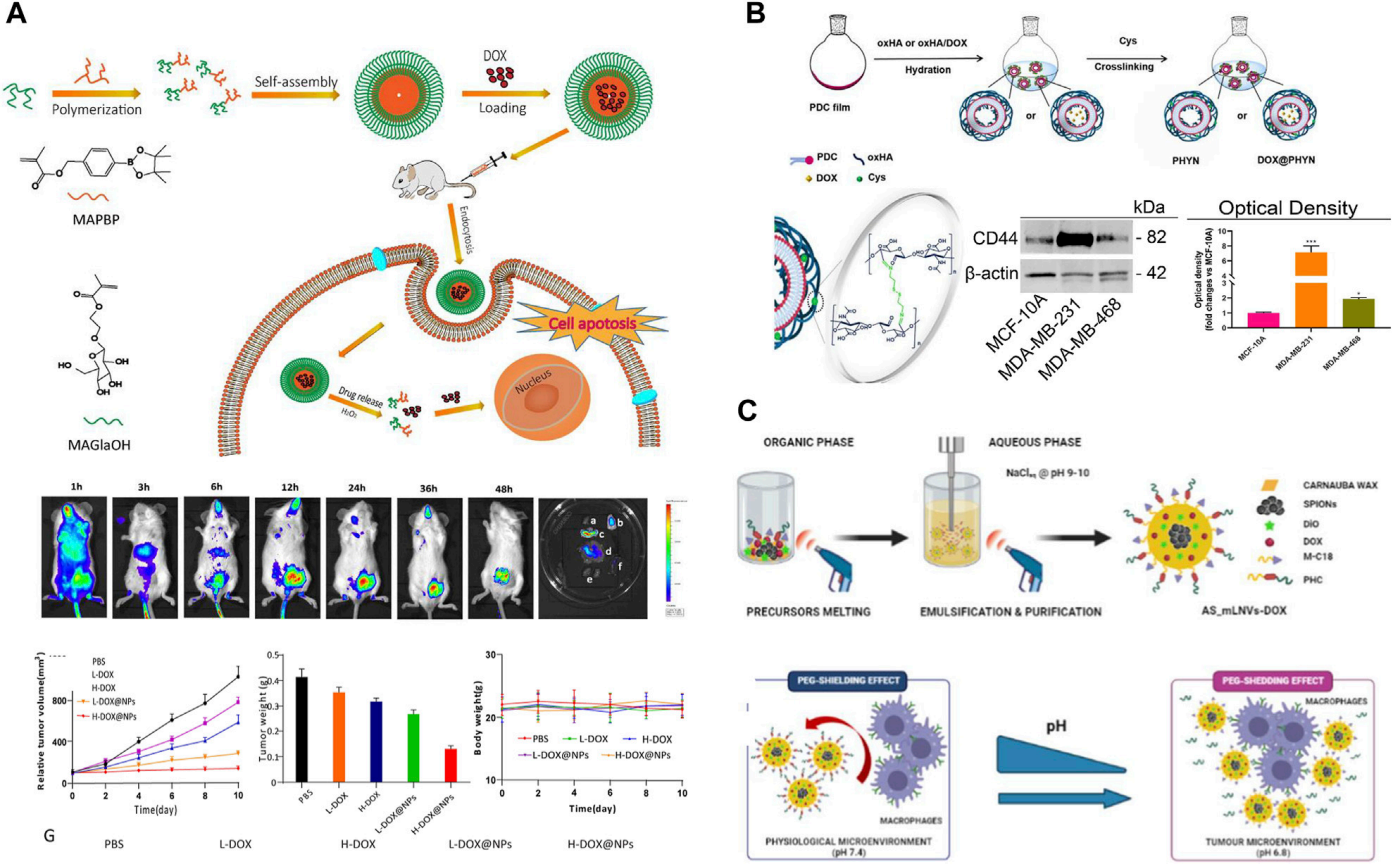
**(A)** Schematic diagram of the preparation and biodistribution of ROS responsiveness DOX@NPs and evaluation of its antitumor activity (Zhou et al., 2023). Copyright 2023. Reproduced with permission from Taylor and Francis. **(B)** Preparation of pH/Redox responsive DOX@PHYN nanoparticles and evaluation of their active targeting level (Curcio et al., 2022). Copyright 2022. Reproduced with permission from mdpi.com. **(C)** Schematic of magnetic nanoparticles AS-mLNVs-DOX with active targeting and pH responsiveness (Scialla et al., 2023). Copyright 2023. Reproduced with permission from Elsevier.

TNBC exhibit high levels of cellular metabolism, proliferation and differentiation, and their physiological characteristics differ significantly from those of normal cells and tissues, mainly in terms of weak acidity, high expression of glutathione (GSH), low oxygenation and overexpression of certain specific enzymes (Mukhopadhyay, 2021; Xiao and Yu, 2021; Gaharwar and Singh, 2023). Thus, the researchers functionalized the nanoparticles with tumor microenvironment responsiveness and specific targeting, which can inhibit the metastasis and invasion of TNBC. It has been found that nano-drug carriers modified by galactose (Gal), phosphatidylcholine bilayer (PDC) and mannose ligand can specifically target the receptors on the surface of TNBC and exert targeting effects. For example, Su et al. (Su et al., 2023) firstly constructed M-mDOX@SiO_2_ nanoparticles with specific targeting of MDA-MB-231 cell surface receptors by coupling DOX and SiO_2_ via pH-responsive dynamic bonding, and then modifying the surface silane with galactoside. The release of DOX from M-mDOX@SiO_2_ showed a strong pH dependence under different acidic media. Fluorescence experiments demonstrated that M-mDOX@SiO_2_ specifically binds to the overexpressed receptors on the cell surface and exerts a targeting effect. The system showed great cytotoxicity to TNBC cells, and almost negligible cytotoxicity to normal cells. Animal experiments demonstrated that the system had potent pro-apoptotic, anti-metastatic and anti-angiogenic effects, thus exerting potent TNBC efficacy. Zhou et al. (Zhou et al., 2023) developed a ROS-responsive chemo-nanoparticles from galactose-conjugated phenylboronic acid for effective targeted delivery of DOX (DOX@NPs). It was found that DOX could be rapidly released under 1 mM H_2_O_2_ medium conditions, and its cumulative release of DOX for 24 h could reach 60%. The DOX nanoparticles with targeted modification significantly increased the uptake of DOX by 4T1 cells and MDA-MB-231 cells, the cellular uptake was 2-fold and 3-fold of that of the control group, respectively. Cytotoxicity experiments showed that the inhibitory effect of DOX@NPs exhibited a strong concentration-dependent effect, the DOX@NPs reduced the IC50 value by about 2-fold compared with free DOX, which was attributed to the great intracellular internalization and ROS-responsive drug release properties of DOX@NPs. *In vivo* experiments indicated that DOX@NPs exhibited higher tumor accumulation in 4T1 *in situ* breast cancer xenografts, achieving targeted delivery and tumor inhibition of DOX (Figure 2A). Curcio et al. (Curcio et al., 2022) constructed an oxidized hyaluronic acid HA (oxHA) crosslinked by phosphatidylcholine bilayer (PDC) and oxidized disulfide bonding (cystamine hydrochloride-Cys), further loaded with DOX to have an active targeting of CD44 receptor (DOX@PHYN). The nanoparticles were modified by PDC to specifically recognize the CD44 receptor on the surface of TNBC cells, which were mainly manifested by higher uptake in MDA-MB-231 cells, while negligible in the normal MCF-10A cell line. Due to the fracture of the dynamic build in the system, in a medium with pH 5.4 and 10.0 mM GSH, the cumulative release of DOX could be as high as 85% within 2 h. In addition, the inhibitory effect of DOX@PHYN on MDA-MB-468 and MDA-MB-231 cells showed a strong time-dependence (Figure 2B). In conclusion, a nanoparticle delivery system capable of actively targeting TNBC as well as responding to both pH/GSH was a smart and promising therapeutic modality for TNBC.

Magnetic nanoparticles (MNPs) it can not only target tumor cells through external magnets, but also serve as a contrast agent for magnetic resonance imaging (MRI). Meanwhile, magnetic carrier drug delivery solves the problem of insolubility of hydrophobic drugs in the blood medium. Therefore, it has received wide concern from researchers. Markhulia et al. (Markhulia et al., 2023) synthesized a superparamagnetic iron oxide nanoparticles delivering DOX by chemical co-precipitation. The system maintains favorable sedimentation stability under physiological pH conditions, and DOX was rapidly released in the presence of ROS. The results of MTT assay showed that the nanoparticles displayed enhanced cytotoxicity against 4T1 and MDA-MB-468 cells and synergistically inhibited cancer cell growth and proliferation. In addition, the combination of drug and magnetic nanoparticles showed good tumor targeting and tissue penetration. This allows for better dose optimization, mitigation of side effects and enhanced inhibition of TNBC cell lines. The reason for this was that iron-containing nanoparticles enhance intracellular ROS production and thus DOX-induced apoptosis in triple positive breast cancer cells. Scialla et al. (Scialla et al., 2023) successfully synthesized magnetic nanoparticles (AS-mLNVs-DOX) with great stability and biocompatibility by one-pot method (Figure 2C). The nanoparticles constructed were modified by a combination of two molecules with complementary functions: 1) mannose ligand (macrophage-targeting); 2) Geranylgeranylic acid sensitivity detachable polyethylene glycol (PEG) molecules (specificity), which endowed the system with excellent pH responsiveness and targeting properties. It was revealed that at physiological pH 7.4, the “shielding” effect of the PEG fragments prevented the mannose receptor on the macrophage surface from recognizing AS-mLNVs-DOX, while DOX could not be released. On the contrary, at the acidic pH of 6.8 in the tumor microenvironment, the acid-sensitive stilbene bonds and PEG chains were broken, which led to the recognition of the mannose receptor targeting the specific receptor on the surface of the TAM, then facilitated the endocytosis of AS-mLNVs-DOX by the TAM, finally, the DOX was successfully targeted to the tumor site. The preferential accumulation of the nanoparticles in mouse tumors carrying M-Wnt tumors did not show significant damage in mouse organs, especially in the heart, compared to free DOX. Thus, the AS-mLNVs-DOX nanoparticles were effective in inhibiting tumor growth and significantly reduced off-target toxicity. In addition, the system can also be used as a contrast agent for T2 in magnetic resonance imaging, As a heat source in magnetic thermotherapy could TNBC’s progress in real time. Therefore, the magnetic nanoparticles with multiple functions could be a therapeutic diagnostic modality for the treatment of TNBC, which could meet the need for more precise and personalized treatment.

### 2.2 Liposomes

Liposomes are hydrophilic bilayer structures formed by phospholipids and cholesterol, which have a high degree of biocompatibility and biodegradability due to their structural similarity to cell membranes. Therefore, DOX is physically encapsulated inside liposomes, which not only protects DOX from enzymatic degradation before it reaches the lesion site, but also improves its stability and reduces its toxicity to optimize the dosage of the drug and to achieve a better therapeutic effect (Peng et al., 2022; Qian et al., 2023). In addition, the surface of phospholipid bilayer can be physically or chemically modified with ligands or other functional groups to make the liposomes tissue-targeted, prolonging the effective retention time of liposomes at the lesion site to improve the delivery efficiency (Si et al., 2021; Nel et al., 2023).

**FIGURE 3:**
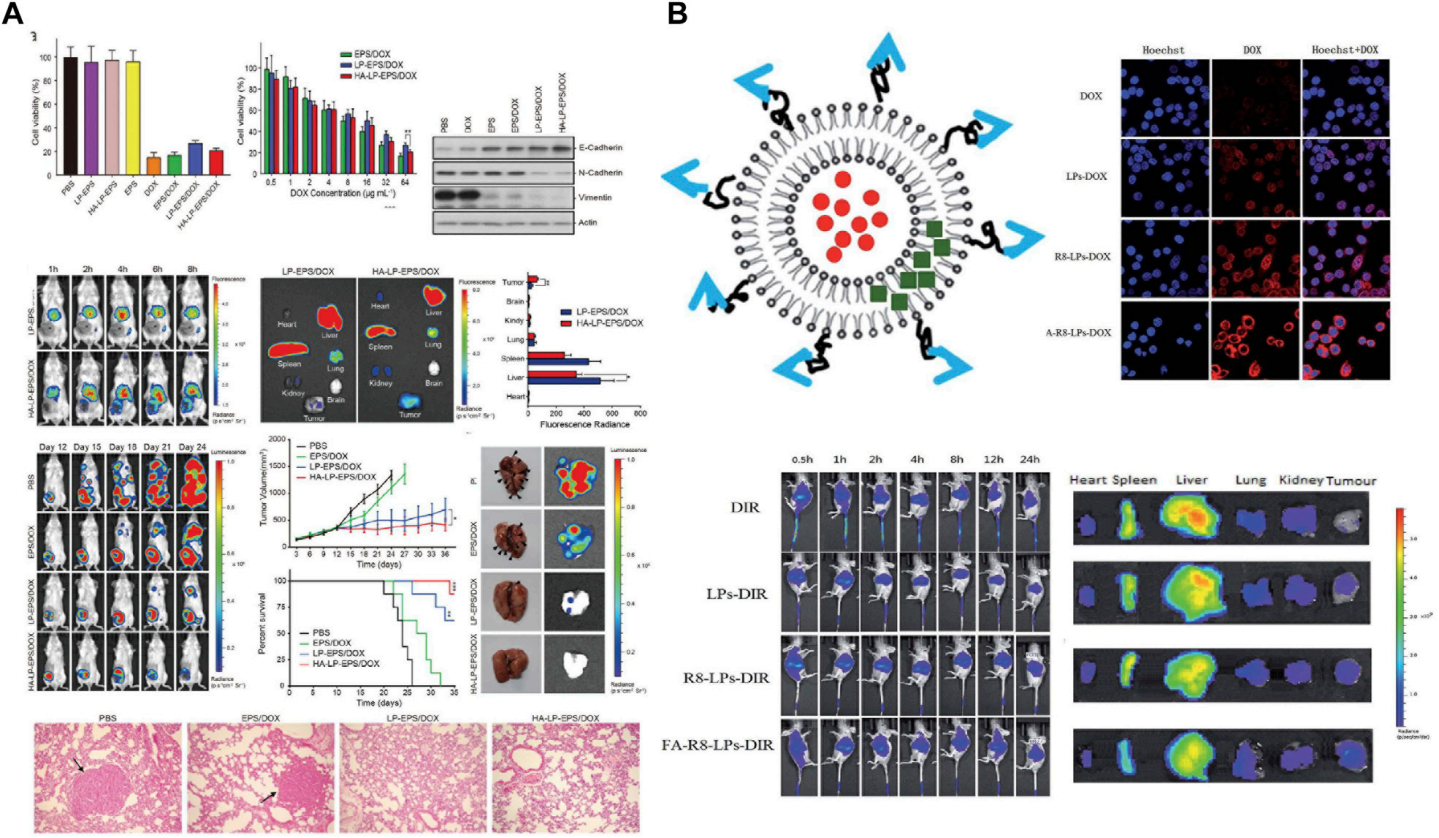
**(A)** Schematic evaluation of the *ex vivo* and *in vivo* activity of specifically targeted liposome CD44-HA-Lip against triple positive breast cancer. Copyright 2022. Reproduced with permission from Wiley Online Library (Dong et al., 2022). **(B)** Schematic representation of the preparation of FA-R8-LPs@DOX/AS-IV liposomes and their *in vitro* antitumor evaluation (Yue et al., 2020). Copyright 2020. Reproduced with permission from Elsevier.

The liposomal Doxil (also known as Myocet) was launched in 1995. A retrospective analysis of multiple clinical trials found that in patients with metastatic triple positive breast cancer who had received prior adjuvant therapy with Doxil, Doxil had a significantly higher anti-tumor response rate and significantly lower cardiotoxicity compared to conventional DOX (Aloss and Hamar, 2023). Since DOX is a classical antitumor chemotherapeutic agent, toxic responses are to be expected. The efficacy of Doxil was verified to be superior to that of conventional DOX through Phase III trials. The full global launch of Doxil ushered in the era of liposomal dosage forms. As a result, researchers are committed to the study of novel formulations of DOX liposome. DOX liposomes hydrochloride (Doxil) is the most marketed and technologically mature variety. Si et al. (Si et al., 2021) employed Stealth technology to increase the resistance of the liposomes at their internal sites by incorporating polyethylene glycol derivatives. High hydrophilicity and flexibility interfere with the hydrophobic interactions between liposomes and plasma proteins. The uptake of liposomes by the reticuloendothelial system (RES) is reduced, which in turn enables the long-circulating function of doxil *in vivo*. Although the passive targeted delivery system is delivered to different sites depending on different sizes, precise targeting cannot be realized. The surface functionalization of DOX liposomes to construct a novel nanodelivery system of liposomes with active targeting ability has drawn much focus in research. Hyaluronic acid (HA), as a natural polysaccharide material with great biocompatibility and low immunogenicity, can target CD44 ligand with superb targeting ability. Dong et al. (Dong et al., 2022) constructed a co-delivery system (HA-Lip-EPS/DOX liposome) with a stable negative charge by loading both epoprostenol (EPS) and DOX with a specific targeting liposome (CD44-HA-Lip) obtained by simple modification of HA (Figure 3A). It was found that the introduction of HA negative charge on the liposome surface could reduce the adsorption of proteins in the blood, thus prolonging the half-life in circulation. *In vitro* experiments showed that the system was well absorbed by cells and inhibited the EMT of TNBC cells as well as reduced the proportion of cells with tumor stem cell (CSC) characteristics. In 4T1 mouse model, the system could deliver both EPS and DOX in tumor tissues with less distribution in other tissues and organs. It also significantly inhibited TNBC tumor growth and epithelial-mesenchymal transition. The system not only also showed the strongest anti-tumor effect among all liposome preparation groups, but also significantly reduced the number of lung metastases and prolonged the survival of mice. Further studies revealed that EPS inhibited the activation of AKR1B1, EMT, and suppressed the CSC properties, tumorigenicity, and metastasis of TNBCs, while the co-delivered DOX effectively killed tumor cells.

Although DOX liposomes can improve anti-TNBC efficiency by reducing tumor metastasis, the dense tumor extracellular matrix makes nanomedicines poorly permeable. To improve it, Yang et al. (Yang et al., 2022) constructed a macrophage-liposome complex (MA-DOX-Lip) by using DSPE-PEG-STA-modified macrophages, DSPE-PEG-Biotin, and DOX. It was found that the constructed MA-DOX-Lip system, which had remarkably low cytotoxicity, no effect on macrophage motility and on the composition of the cell membrane as well as on cellular functions. As the system could successfully localize to the tumor site of TNBC model mice and release DOX deep into the tumor tissue. The tumor inhibition rate was as high as 99.5% at the 21st day of treatment, and the average survival time of mice could be 30 days. Particularly, the system could release more intense fluorescent molecules that penetrate more deeply into tumor tissues to exert anti-TNBC effects. Thus, the delivery system is expected to serve as a novel approach for the treatment of TNBC. Then, in order to solve the resistance problem of DOX during chemotherapy of TNBC, Yue et al. (Yue et al., 2020) found that astragaloside IV (AS-IV) had a promising reversal of DOX resistance, based on which, the group constructed a liposome-targeted co delivery system FA-R8-LPs@DOX/AS-IV (Figure 3B). The incorporation of FA and R8 effectively improved the tumor targeting and cellular uptake ability of liposomes. The results of cytotoxicity assay showed that FA-R8-LPs significantly inhibited the proliferation of DOX-resistant cell line MDA-MB-231/DOX. At the same time. The system markedly inhibited tumor growth and overcame DOX resistance to exert a excellent anti-tumor effect in TNBC nude mouse tumor model.

### 2.3 Micelles

Micelles are thermodynamically stable colloids formed by self-assembly of amphiphilic polymers. The particle size of micelles is generally in the range of 10–100 nm, they are not easily recognized and captured by the endothelial reticuloendothelial system (RES) in the blood circulatory system, and the nanocarrier system can be stable and long-lasting in the bloodstream. At the same time, it can achieve passive targeting to the tumor site through the EPR effect (Atanase, 2021; Nel et al., 2023). The inner core of the micelles encapsulates a large amount of hydrophobic drug, which greatly improves drug solubility, while the hydrophilic outer shell acts as a barrier to avoid micelle aggregation. In addition, after proper functionalization of its core and shell, the hydrophilic shell can interact with biological components, which affected pharmacokinetics and drug distribution while the lipophilic core can be used to encapsulate the drug and release it, the DOX can be better loaded in the core of micelles (Qian et al., 2023). Therefure, micelles have a bright future of application in drug delivery.

**FIGURE 4:**
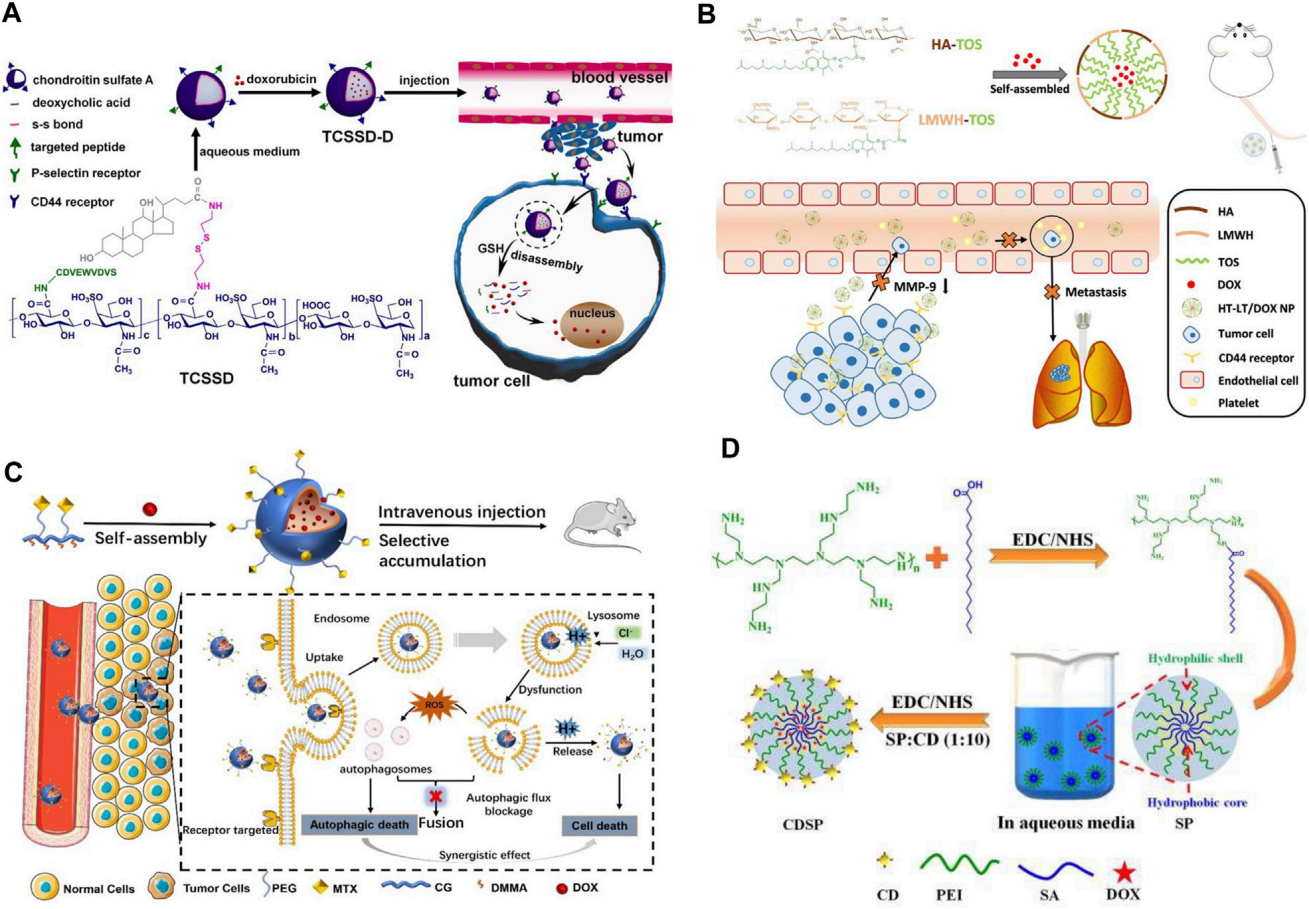
**(A)** Schematic representation of the preparation of redox-responsive C-TCSSD-DOX micelles, *in vivo* cellular internalization, and drug release in tumor cells (Yu J et al., 2023). Copyright 2023. Reproduced with permission from Elsevier. **(B)** Schematic illustration of the preparation of LMWH-TOS micelles and its anti-tumor action mechanism (Yang et al., 2020). Copyright 2020. Reproduced with permission from Elsevier. **(C)** Schematic diagram of the preparation of MTX-PEG@CG/DMMA@DOX micelles and its interference with autophagic flux and drug delivery (Cao et al., 2023). Copyright 2022, Reproduced with permission from Springer. **(D)** Schematic representation of the preparation and evaluation of the antitumor effect of amphiphilic nano-DOX-CDSP-25 micelles (Sarkar et al., 2021). Copyright 2020. Reproduced with permission from Elsevier.

Designing nanomicelles with targeting effect can solve the problem of toxic side effects of DOX on normal tissues and cells in the treatment of TNBC. For example, Yu et al. (Yu J et al., 2023) prepared a high drug loading micelle (C-TCSSD-DOX) with a targeted peptide (CDVEWVDVS) grafted with chondroitin sulfate A-ss-deoxycholic acid (TCSSD) copolymer and loaded with DOX (Figure 4A). The DOX in the drug-loaded micelles was rapidly released in a release medium containing 20 mM of GSH, the cumulative release rate could reach 73.8% ± 3.11% at 96 h. The reason was the introduction of disulfide bonds in the system, under the stimulation of high GSH in the tumor site, the disulfide bonds were broken, the rapid release of DOX in the tumor cells exerted the anti-tumor effect. It was also found that with the introduction of the targeting peptide, the system was able to effectively target CD44 and P-selectin receptors both *in vivo* and *ex vivo*. Thus, the C-TCSSD-DOX micelle group exhibited lower cytotoxicity, higher cellular uptake and the strongest tumor growth inhibition. Yang et al. (Yang et al., 2020) constructed a stable and targeted heterogeneous micelle (HT-LT) with hyaluronic acid-d-α-tocopheryl succinate (HA-TOS, HT) and low molecular weight heparin-TOS (LMWH-TOS, LT) by simple mixing. It was able to achieve up to 80% loading of DOX (Figure 4B). *In vivo* and *in vitro* experiments confirmed that HT-LT@DOX exhibited strong targeting ability in both 4T1 models, which was attributed to the binding of HA to CD44 receptor that recognizes specific tumor cells. In addition, HT-LT@DOX acted at different stages of invasion-metastasis and inhibited the migration and invasion of tumor cells.

In order to solve the problems of cardiotoxicity and easy autophagy off-target of DOX in the treatment of TNBC chemotherapy. Cao et al. (Cao et al., 2023), by modifying methotrexate with PEG (MTX-PEG), coupling CG/DMMA, loading DOX, prepared a MTX-CG@DOX micelles (Figure 4C). Under the buffer medium of pH 5.4, the cumulative release of DOX from micelles could be as high as 85.6% within 48 h, while it was only 25.7% at pH 7.4. Fluorescence staining experiments found that DOX micellar system could cross the cell membrane through the endocytosis pathway after MTX could bind specifically with folate receptor, which could alleviate the accumulation and damage of DOX in normal organs. HE staining experiments showed that there was no obvious necrosis, edema or inflammatory infiltration of DOX micellar system in the major organs. In addition, DOX micelles could effectively promote the accumulation of autophagosomes in tumor cells, interfere with the degradation process of autophagic flow, and jointly induce autophagic death of TNBC cells. The targeted delivery of DOX is coupled with the simultaneous bioimaging of TNBC, which allows real-time monitoring of TNBC growth, metastasis and invasion. This novel micellar delivery system is expected to be further translated to clinical trials. Sarkar et al. (Sarkar et al., 2021) pioneered the construction of amphiphilic nanomicelles (DOX-CDSP-25) from stearic acid (SA) and polyethyleneimine (PEI) and folic acid-derived carbon dots (CDSP) (Figure 4D). Cytotoxicity experiments revealed that DOX-CDSP-25 exhibited similar cytotoxicity against TNBC at a concentration of only 1.0 μg/mL to that of free DOX (IC50 of about 10 μg/mL). The nanomicelles could be successfully endocytosed and ingested by MDA-MB-231 cells, which could be attributed to the specific uptake of CDSP-25-mediated receptors by MDA-MB-231 cells. After 24 h of DOX-CDSP-25 treatment of MDA-MB-231 cells, a large number of membrane vesicles and apoptotic vesicles appeared, the membrane integrity of MDA-MB-231 cells was lost, which indicated that the nanomicelles were able to efficiently deliver DOX into the tumor cells, exerting its anti-tumor effects through apoptosis.

### 2.4 Nanogels

Nanogels (NGs) have gained much interests as drug carriers due to the advantages of both hydrogels and nanoparticles, NGs are versatile hydrophilic drug delivery platforms for targeted release of hydrophobic drugs and biomolecules (e.g., proteins or nucleic acids) (Cuggino et al., 2019; Kass and Nguyen, 2022). Due to their inherent porosity, NGs also have an excellent high drug-carrying capacity compared to liposomes or polymeric micelles, which can accommodate more than 30% wt or more of drugs. More importantly, hydrogels can respond rapidly to external environmental (e.g., ionic strength, pH, or temperature) stimuli through swelling, shrinkage, or sol-gel phase transition (Chen et al., 2022; Zhang Y et al., 2022). Thus, it is of great significance to construct multifunctional DOX nanogels for the treatment of TNBC.

**FIGURE 5:**
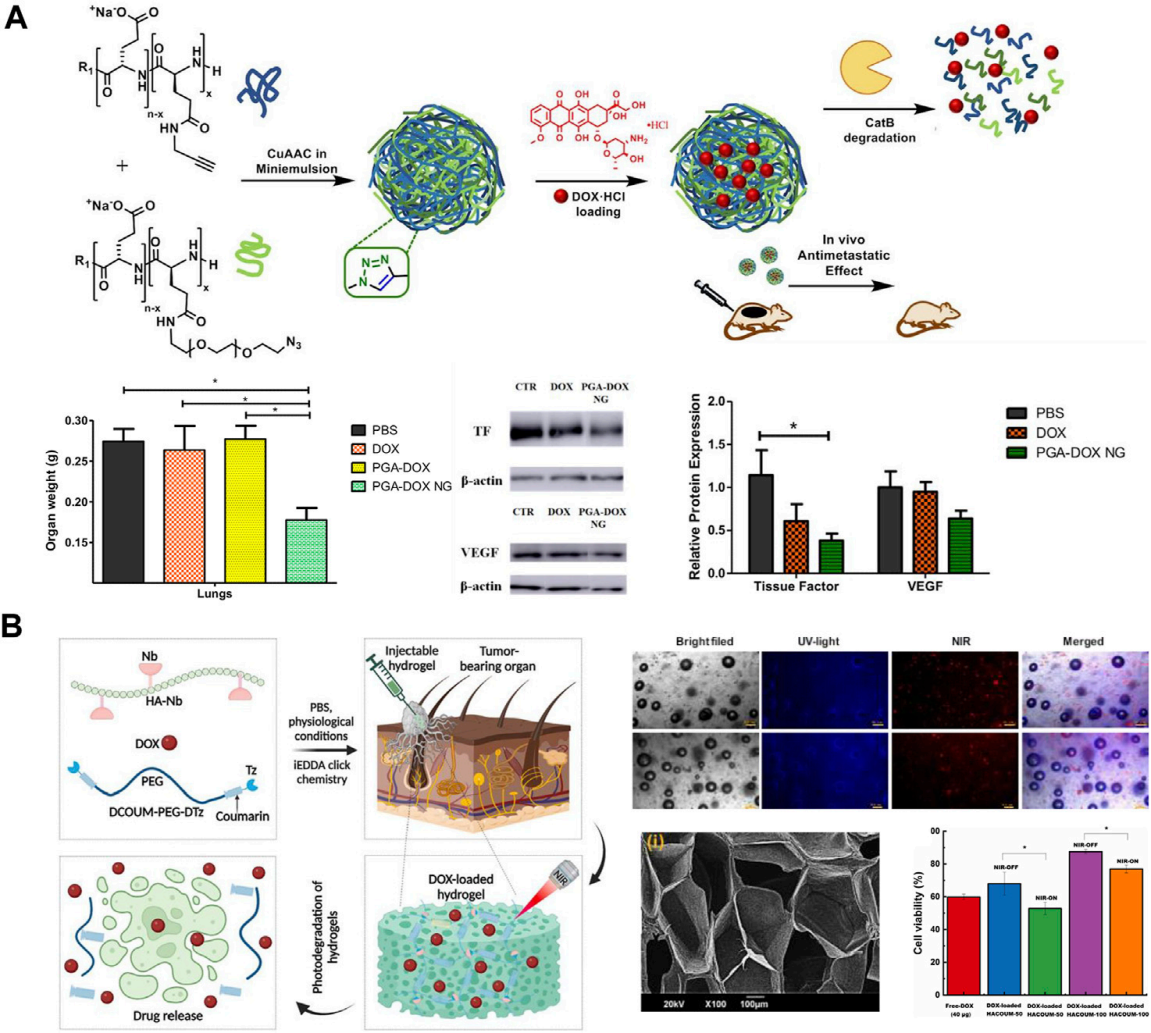
**(A)** Preparation of PGA-DOX NGs nanogels and its mechanism of action (Duro-Castano et al., 2021). Copyright 2023. Reproduced with permission from Elsevier. **(B)** Synthesis of photodegradable hydrogel and schematic diagram of its drug release mechanism (Gulfam et al., 2023). Copyright 2020. Reproduced with permission from Elsevier.

Researchers have used injectable polyamino acid nanogels (NGs) as a multifunctional hydrophilic drug delivery platform for the treatment of TNBC metastases. Duro-Castano et al. (Duro-Castano et al., 2021) constructed an injectable nanogel delivery system with excellent biocompatibility by using polyglutamic acid (PGA) and loading the chemotherapeutic drug DOX (PGA-DOX NGs) (Figure 5A). It was observed that the cumulative release of PGA-DOX NGs could be as high as 80.0% for 25 h under Cathepsin B conditions, while it was not higher than 5% in PBS pH 7.4 medium. Compared with free DOX, this delivery system exhibited higher anti-migration inhibition and also promoted 4T1 cell aggregation toward G2/M phase and hindered 4T1 cell division. In addition, in the *in situ* mouse TNBC model with spontaneous metastasis, the injectable PGA-DOX NGs not only significantly reduced the volume and weight of primary tumors in TNBC mice, but also showed strong anti-metastatic ability in lungs and axillary lymph nodes, with an anti-metastatic rate of up to 80%, surprisingly, the system has almost no toxic side effects on normal tissues and organs of mice.

For example, Xie et al. (Xie et al., 2017) synthesized a self-repairing heat-sensitive magnetic gel (DD-Fe@DOX) with docetaxel (DTX), bifunctional polyethylene glycol (DF-PEG-DF), iron oxide magnetic nanoparticles (Fe_3_O_4_ NPs), and DOX. Stimulation of iron oxide with magneto-thermal therapy induced the release of DOX and DTX, exerting a synergistic anti-TNBC effect. *In vitro* release experiments demonstrated that the system could achieve a slow-release release of DOX and DTX for up to 30 days. After co-culturing L929 cells with magnetic hydrogel 3D for 48 h, their cell survival rate could be as high as 99.3%, indicating that the self-repairing magnetic hydrogel could be used as an extracellular matrix mimic and could provide a good microenvironment for cell survival. However, the inhibitory effect of DD-Fe@DOX hydrogel on TNBC showed good time-dependent and concentration-dependent effects, and the cell survival rate was only 5.6% after 48 h of incubation, and the DD-Fe@DOX delivery system showed a more obvious effect on the synergistic anti-tumor activity of triple positive breast cancer cell lines and on the reduction of tumor size.

Injectable gels can be administered in a locally targeted and minimally invasive manner through a narrow syringe without the need for invasive procedures, allowing for minimal-harm interventions in patients with TNBC. Gulfam et al. (Gulfam et al., 2023) prepared a gel (HACOUM) for delivery of DOX using hypocretin (NB)-functionalized HA with terminal tetrazine (TZ) modified coumarin analogs as cross-linking agents (Figure 5B). It was found that HACOUM-100 as a model could be successfully extruded through a 25-gauge injection, indicating that the gel has great injectability and can accurately inject drugs into diseased tissues or organs. Standard metabolic test experiments found that even in the hydrogel (HACOUM-100) formed by high concentration of HA-NB (2000 μg/mL) and cross-linking agent (1,000 μg/mL), then co-cultivated with HEK-293 cells for 48 h, the cell survival rate was still above 90%. BT-20 cells as model cells, it was found that the hydrogel released DOX at a slow rate in the absence of near-infrared light, and its antitumor activity was low, whereas the coumarin ester portion of the hydrogel was partially cleaved by near-infrared radiation, which loosened the unchaining of the hydrogel network, and the DOX was rapidly released, showing high antitumor activity. More surprisingly, the gel can be excited in the visible spectral range and can emit green to red fluorescence, which can be applied to bioimaging for TNBC diagnosis. In addition, the good biocompatibility and degradability of the hydrogel expanded the application of DOX. In addition, the great biocompatibility and degradability of hydrogels can reduce the toxic side effects of DOX, Onder et al. (Onder et al., 2023) prepared poly (acrylic acid)-2-hydroxyethyl methacrylate/rutin (RTN hydrogel) by using the polyphenolic compound rutin as a natural cross-linking agent. This RTN hydrogel was great biocompatible, with a cell survival rate of up to 95% in normal cell lines, and its biodegradable and antioxidant properties had significant inhibitory effects on the proliferation and migration of TNBC cell lines.

In addition, the three-dimensional structure of the nanogel enables slow and controlled release of DOX. Zhang et al. (Zhang et al., 2020) synthesized nanogels with pH and redox response by Schiff base reaction using dextran aldehyde, cysteine and DOX. In the tumor microenvironment’s (TME) medium (pH 5.4 + 10 mM GSH), DOX in the nanogels was rapidly released in the first 24 h and its cumulative release was up to 90%. In contrast, its cumulative release did not exceed 20% in normal physiological medium. Fluorescence staining experiments revealed that the system could rapidly endocytose DOX into the interior of TNBC cell lines, effectively killing 3T3 and MDA-MB-231 cells. Compared with topical delivery, this system of DOX was sustainably released for up to 10 days from this hydrogel injected in a TNBC mouse model, and the nanogel was able to accumulate at the tumor site after 48 h of systemic administration, with tumor growth ceasing after 3 days. More surprisingly, the gel delivery system could better inhibit the metastasis of TNBC cells to other tissues and organs. However, the slow and controlled release continuous delivery could further improve the clinical efficacy, but the accumulation of nanogel in the liver and spleen was unavoidable.

### 2.5 Dendrimers

Dendrimers are monodisperse polymers with a highly branched structure consisting of an inner core, a polymer backbone and side chains of dendritic units (Sherje et al., 2018; Kesharwani et al., 2022). The most important feature of the dendritic macromolecule is its built-in cavity structure, which protects DOX from enzymatic digestion and hydrolysis and reduces rapid clearance by the immune system. It can also significantly prolong the half-life of the drug in the body and minimize the frequency of drug administration. In addition, the surface of dendrimers is densely covered with various functional groups, which can specifically bind to target cells after modification, realizing the targeted delivery of DOX and enhancing the chemotherapeutic effect of TNBC (Mukherjee et al., 2022; Dhull et al., 2023). Thus, based on these properties of dendrimers, they have led to the development of novel drug delivery technologies.

Poly (amide-based amine) (PAMAM) are highly branched macromolecules with numerous reactive amine groups on the surface, their unique properties, PAMAM dendrimers are prone to a wide range of applications in drug delivery (Li et al., 2018; Surekha et al., 2021). Guo et al. (Guo et al., 2019) synthesized a highly stable dendritic polymer (HA-PAMAM-Pt@DOX) by coupling DOX and cisplatin (Pt), with HA as a target group and polyamide (PAMAM). Its Pt and DOX drug loading efficiency was 13.6% and 18.3%, respectively. Endocytosis experimental studies revealed that the system could enter the cell via a lysosomal-mediated pathway, it also showed a significant time-dependence. The cytotoxicity experiment found that HA-PAMAM-Pt@DOX group showed higher anti-cancer activity on MCF-7 and MDA-MB-231 cells only at 1.5 μg/mL. *In vivo* tissue distribution studies showed that HA-PAMAM-Pt@DOX significantly increased drug accumulation in tumor tissues with little damage to the heart and kidneys. Meanwhile, it was revealed that the metal dendritic polymer had strong anticancer cytotoxicity and accumulated higher in tumor tissues than in other organs, with a more remarkable anti-TNBC effect.

**FIGURE 6:**
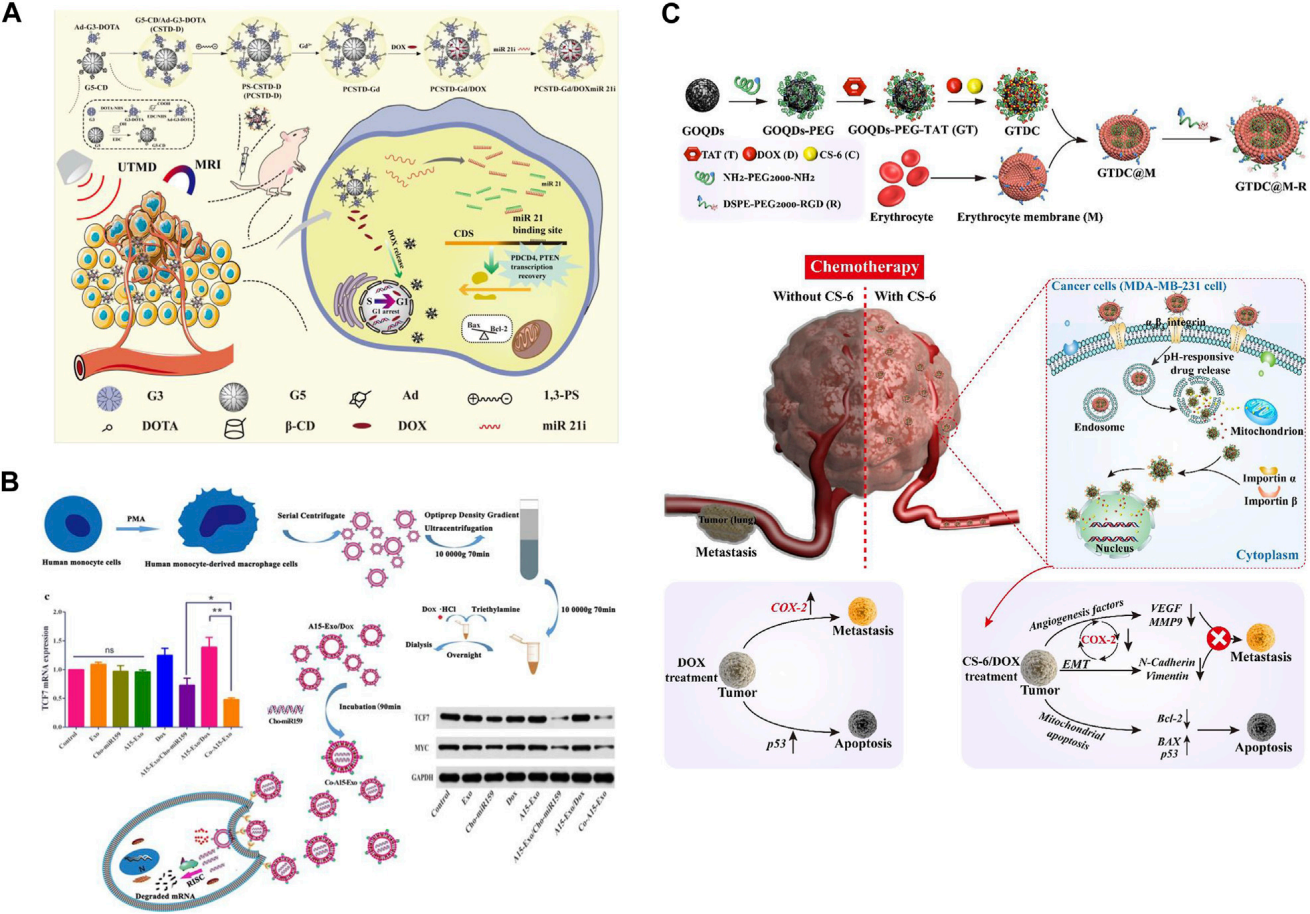
**(A)** Schematic diagram of the preparation and evaluation of anti-tumor effects of multifunctional CSTD nano-delivery system (Gong J et al., 2023). Copyright 2023. Reproduced with permission from pubs.rsc.org. **(B)** Schematic diagram of dual-sensitive nanogel for sustained DOX delivery (Gong et al., 2019). Copyright 2020. Reproduced with permission from Europe PMC. **(C)** Schematic diagram of the preparation and evaluation of anti-tumor effects of multifunctional CSTD nano-delivery system (Fan et al., 2020). Copyright 2023. Reproduced with permission from Elsevier.

Ruthenium (Ru-II) (Rahman et al., 2018; Chen et al., 2020) and Gadolinium (Gd-III) (Siribbal et al., 2022; Zhang et al., 2023) are metals with different degrees of oxidation. In living organisms, Ru-II and Gd-II can attach to transferrin and albumin, leading to programmed cancer cell death. Thus, ruthenium carbosilane-based and gadolinium dendrimers are of great interest to researchers as drug carriers for delivery of TNBC. Michlewska et al. (Michlewska et al., 2023) synthesized a carbosilane metal dendritic polymer (CRD13) and loaded with DOX, 5-fluorouracil (5-Fu), and methotrexate (MTX) drugs to form metal dendrimeric complexes (CRD13@DOX). It was found that CRD13 metal dendrimer complex could encapsulate DOX, 5-Fu, and MTX up to 80%. The results of MMT experiments showed that the inhibitory effect of CRD13@DOX was significantly higher than that of DOX on MDA-MB-231 cancer cells, This may be due to the interaction of metal Ru with tumor cell DNA. *In vivo* experiments demonstrated that when CRD13@DOX was injected intravenously, it could cause CRD13@DOX to accumulate at the site of the lesion and significantly reduce the weight and size of tumors in TNBC mice *in vivo*. Gong et al. (Gong J et al., 2023) constructed a metal dendritic polymer (CSTD) with *ß*-cyclodextrin and modified Gd(III), co-delivered MicroRNA 21 inhibitor (MiR 21i) and DOX, used ultrasound-enhanced treatment of *in situ* TNBC model (Figure 6A). Its large size of the internal cavity structure allowed DOX to achieve an encapsulation rate of up to 84.0%. miR 21i had the best transfection efficiency at an N/P ratio of 15 for optimal tumor targeting compared to the control. Its superb tumor permeability and penetration allowed T1-weighted MR imaging of *in situ* TNBC models, it also showed significant anti-tumor effects and liver metastasis inhibition.

In order to enhance the chemotherapeutic effect of DOX and reduce its drug resistance, chemotherapy combined with gene therapy has made significant progress in the field of TNBC (Maheshwari et al., 2021; Paskeh et al., 2022). In gene therapy, small interfering RNA (siRNA) has become a hotspot for research. In gene therapy, siRNA has become a research hotspot. siRNA molecules can bind to target genes and use complementary sequences to degrade target messenger RNAs, thus silencing various target genes related to poor tumor prognosis and exerting therapeutic effects. Kesharwani et al. (Kesharwani et al., 2023) co-delivered the chemotherapeutic drug DOX, the chemoprotectant lycopene (LCP), and siRNA genes using polyamide (PAMAM) dendritic polymers as a carrier. It was found that the carrier could stably encapsulate DOX, LCP, and siRNA, which could protect them from enzymatic degradation and hydrolysis, reduce rapid clearance by the immune system, and prolong the duration of action at the tumor lesion site. PAMAM-loaded (DOX of 10 µM) inhibited the proliferation of TNBC cells by 80% compared to the carrier-free system. In a TNBC xenograft model, the combination of silencing of the survival protein siRNA and DOX significantly inhibited TNBC cell growth and metastasis. The combination of drug delivery systems reduced the cardiotoxicity and drug resistance of DOX. Jain A et al. (Jain et al., 2019) delivered siRNA-PLK1 and DOX with PAMAM dendrimer for the treatment of TNBC. It was found that the loaded drug could be stabilized in the inner lumen of the dendrimer, which was better able to successfully block the MDA-MB-231 and MCF-7 cells with G1. It was also able to strikingly increase the cellular uptake of siRNA-PLK1 to exert an anti-TNBC effect. Liu et al. (Liu et al., 2019) constructed a novel dendrimer nanodelivery system (EBP-1-PAMAM-TAT@DOX) by attaching an EGFR-targeting peptide (EBP-1) to a PAMAM dendrimer, which was conjugated with a cell-penetrating peptide of a transcriptional activator of transcription (TAT) and loaded with DOX. Compared with free DOX and monofunctional carriers, this system markedly improved drug accumulation at tumor sites *in vivo*, resulting in tumor growth inhibition and prolonged survival.

### 2.6 Exosomes

Exosome is a kind of microvesicles with a diameter of 30–150 nm, which is collectively called extracellular microvesicles together with microvesicles and apoptotic vesicles. Exosomes have a typical phospholipid bilayer membrane structure and low immunogenicity, which can cross the blood-brain and placental barriers more efficiently. It can deliver biological information such as proteins and genetic material from source cells to recipient cells, which can change their physiological and pathological states, as well as activate the next signaling pathway (Wang et al., 2021; Kar et al., 2023). Thus, exosomes are able to deliver various drugs, antigens, and molecules to TNBCs and interact with them via their cell surface markers, allowing TNBCs to apoptose and promote anti-tumor immune responses. This approach has improved the treatment of TNBC patients, increasing their survival and quality of life (Weaver et al., 2022). Thus exosomes as nano-drug carriers for delivery of TNBC have received much attention from researchers.

Exosomes from different sources have diverse therapeutic effects in the treatment of TNBC. For example, Gong et al. (Gong et al., 2019) constructed an exosome co-delivery system (Cho-miRN-Exo@DOX) by incubating cholesterol-modified miRNAs (Cho-miRNAs) coloaded into exosomes enriched with A15 protein. It found that A15-Exo (1 µg) incubated with DOX (200 μg/mL) exhibited the maximum loading (80%) (Figure 6B). The cumulative release of DOX could be as high as 90.5% at pH 5.0 (late intracellular body and lysosomal environment). The fluorescence intensity and uptake (28.26%) and apoptosis rate (47.15%) were significantly higher than those of the control group. A15-Exo bound more efficiently to the integrin αvβ3 on the cell surface of MDA-MB-231 and B16 cells, which greatly enhanced its exosome targeting effect. In addition, it was found that Co-A15-Exo notably downregulated TCF7 protein expression in cells and significantly inhibited tumor cell growth and tumor angiogenesis. In a mouse xenograft tumor model, A15-Exo was able to deliver the carried miRNAs to the tumor tissues in a targeted manner, as a result, the volume and weight of the mouse tumors could be dramatically reduced, the survival rate of the mice could be significantly prolonged, more surprisingly, due to its excellent targeting, the system was virtually non-toxic to the heart.

Li et al. (Li et al., 2020) prepared a C-Met-targeted with macrophage-derived exosome membrane-loaded DOX nanoparticles for targeted chemotherapy of TNBC (Met-Exo-PLGA@DOX). *In vitro* release experiments revealed that the exosome membrane coating acted as a diffusion barrier for DOX, which remarkably prolonged and improved the release of DOX. C-Met-modified exosomes significantly increased the cellular uptake rate and the antitumor efficacy of DOX. Quantitative results of flow cytometry showed that the apoptosis rate of MDA-MB-231 cells was as high as 39.73% after 12 h, which was markedly higher than that of Met-Exo-PLGA (29.27%), PLGA@DOX (11.33%) and DOX (10.58%). *In vivo* experiments showed that Met-Exo-PLGA@DOX systems had a longer circulating lifetime and great tumor inhibition effect. Thus, the use of macrophage-modified exosomes as a promising drug delivery strategy for TNBC therapy. Uslu et al. (Uslu et al., 2022) obtained a PLT-Exo-DOX system by ultracentrifugation of exosomes extracted from platelets and then wrapped with DOX, the DOX encapsulation efficiency of which could be as high as 86.02% ± 6.16%. It was found that the system significantly inhibited the proliferation of the TNBC cell line, it also could significantly block the cells in G1/M phase. In addition, the PLT-Exo-DOX system significantly increased the binding level of Annexin-V and the expression of Bax gene, which further promoted the apoptosis of TNBC cell lines. It indicated that the system constructed by exosome-loaded DOX exhibited great potential in the treatment of TNBC, and also provided new ideas for the new treatment of TNBC.

### 2.7 Other delivery systems

In addition, other novel nanomaterials (e.g., graphene oxide (GO), carbon quantum dots (QDs), carbon nanotubes (CNTs), nanorods, nanodroplets, etc.) have drawn much attention from researchers due to their unique physicochemical properties (Choi et al., 2021). With the properties of graphene oxide (GO) flexibility, electrical conductivity, great biocompatibility and responsiveness to external stimuli. Sahoo et al. (Sahoo et al., 2022) designed a novel remote on-demand drug delivery system. DOX was remotely controlled for on-demand release using low voltage on the GO surface and a handheld cell phone. It was found that DOX was released stably under external voltage and showed potent inhibition of MDA-MB 231. Graphene oxide quantum dots (GOQDs) have high specific surface area, easy surface modification, and excellent fluorescence properties. GOQDs can not only act as drug delivery carriers, but also be used for fluorescent labeling. Fan et al. (Fan et al., 2020) modified GOQDs wrapped with erythrocyte membranes with nucleus-targeted transcriptional transactivator (TAT) peptide (GT NPs), then combined the loading of DOX and Gamabufotalin (CS-6) to co-construct a nano-quantum dot system (GRCD NPs). The system had an optimized targeting capability (Figure 6C). *In vitro* experiments demonstrated that the nanosystem was biocompatible, significantly extended blood circulation time (3-fold longer than GT NPs), effectively enhanced cell and nucleus targeting ability. DOX induced more than 89% apoptosis in TNBC cells when combined with CS-6 at a ratio of 10:1. At the same time, the accumulation of GTDC@M-R NPs at the tumor site was increased by approximately 2-fold compared to pure GTDC NPs. The system also enhanced the anti-TNBC tumor activity and metastatic tendency to the lungs by activated COX-2 and enhanced DOX. More interestingly, the GTDC@M-R NPs system utilized its luminescent properties to enable real-time monitoring of TNBC.

In addition to GO, carbon nanotubes (CNTs) are a promising class of inorganic carbon nanomaterials, where surface grafting of different hydrophilic molecules can modulate the drug-carrying efficiency. In addition, the surface functionalization of CNT nanocarriers endows the system with good targeting ability, which not only improves the cellular uptake efficiency, but also reduces the cytotoxicity by controlling the mode of drug release *in vitro*. Singhai et al. (Singhai et al., 2020) constructed a novel multi-walled carbon nanotube drug-loaded system (α-TOS-HA-MWCNTs@DOX) by modifying multi-walled carbon nanotubes (MWCNTs) with HA and *a*-tocopheryl succinate (α-TOS) and loading DOX. It was found that the MWCT-loaded DOX modified by HA and *a*-TOS exhibited a high cellular uptake rate, its apoptosis rate was also as high as 52.69% ± 4.86%. All results indicated that HA and *a*-TOS could be used as a synergistic, safe, and effective tumor-targeted chemotherapeutic drug DOX for the treatment of TNBC.

Magnetic nanorods not only have high drug loading rate, strong tissue permeability as well as magnetic targeting therapy, they can be used to load a variety of tumor-targeted therapeutic drugs to achieve synergistic treatment of various types of cancer. Lu et al. (Lu et al., 2023) modified Fe3O4 with macrophage membranes to obtain vortex nanorods, which were then loaded with DOX and Zeste enhancer homolog 2 (EZH2 siRNA), and the novel nanomedicine exhibited excellent tissue permeability and tumor accumulation. Cytotoxicity experiments showed that the combination of DOX and EZH2 siRNA had a synergistic inhibitory effect on TNBC cells, and the nanomedicine showed excellent safety after systemic delivery due to the system’s favorable tumor-targeting ability. More importantly, it was shown in a 4T1 mouse xenograft tumor model that magnetic nanorods induced photothermal cell death and increased solid tumor temperature *in vitro* after stimulation with NIR energy, which resulted in irreversible thermal damage to mice after intratumor injection and inhibited TNBC growth.

Furthermore, nanodroplets have the advantages of small particle size, low viscosity, high diffusion and penetration ability as well as high bioavailability, which show great advantages in the treatment of TNBC. Xiao et al. (Xiao et al., 2023) prepared a homogeneous and stable nanodroplet (HA-SS-CMC-ND) containing HA and carboxymethyl chitosan (CMC) to deliver DOX. It was found that the system for DOX released *in vitro* showed great pH and redox response properties. The system also had good biocompatibility and active targeting ability, resulting in a normal cell survival rate higher than 95%, its significantly higher anti-proliferative and anti-migratory abilities against MDA-MB-231 than other controls. The system also can be used as a contrast agent for specific tumor imaging. More surprisingly, the HA-SS-CMC-ND combined ultrasound-targeted microbubbles improved drug aggregation and retention within MDA-MB-231/ADR, which could upregulate the ROS level, scavenge GSH, and promote apoptosis. It also reduced the expression level of P-glycoprotein, inhibited epithelial-mesenchymal transition, and synergistically counteracted multiple drug resistance. This combination strategy showed protective effects against TNBC in both MDA-MB-231/ADR cell and female BALB/c mice (Mice models constructed from 4T1 cells).

## 3 Clinical trial of ICIs combined with DOX for TNBC treatment

In recent years, immunotherapy can effectively prolong the survival of TNBC patients, which is expected to provide more treatment options for TNBC patients (Naimi et al., 2022). Among the immunotherapies for TNBC, the most mature research has been conducted on immune checkpoint inhibitors (ICIs), which can directly block immunosuppressive receptors and improve the cytotoxicity and proliferation of tumor-infiltrating lymphocytes. Currently, the most widely used ICIs are mainly PD-1 and its ligand PD-L1 inhibitors. Although TNBC has a high tumor mutational load and PD-L1 expression, a high number of tumor-infiltrating lymphocytes (TILs), the objective remission rate (ORR), overall survival (OS), and progression-free survival (PFS) of monotherapy with ICIs have failed to be achieved in patients with TNBC (Khan et al., 2023; Yu Y et al., 2023). Atezolizumab, a humanized immunoglobulin IgG1 monoclonal antibody, therapeutically blocks PD-L1 and enhances the extent and quality of tumor-specific T-cell responses, leading to improved antitumor effects (Kwapisz, 2021). Atezolizumab has been shown to have significant anti-tumor effects in preclinical cancer models and in patients with different tumor types. Based on the IMpassion130 study (Emens et al., 2021), the FDA approved Atezolizuma in combination with albumin paclitaxel for the first-line treatment of PD-L1-positive inoperable locally advanced/metastatic TNBC. However, the IMpassion131 trial (Miles et al., 2021) did not replicate these findings, with no improvement in PFS or OS in either the ITT population or the PD-L1-positive population. Following this, the FDA withdrew the indication for Atezolizuma in advanced TNBC. Subsequently, the ALICE trial (Røssevold et al., 2022) investigated the neoadjuvant use of Atezolizumab in combination with polyethylene glycolated liposomal doxorubicin and low-dose cyclophosphamide for the treatment of TNBC. In the compliant population, PFS improved in the Atezo-chemo group (median 4.3 vs. 3.5 months; hazard ratio (HR) = 0.57; FAS (HR = 0.56. 95%, CI 0.33%-95%). The progression-free rate at 15 months was 14.7% (CI 6.4%-30.1%) in the Atezo-chemo group *versus* 0% in the placebo chemotherapy group. To further validate the above clinical trials, a global, prospective, randomized, open-label Phase 3 trial, IMpassion030 (Heather and McArthur, 2019; Saji et al., 2021), further investigated the efficacy, safety, and pharmacokinetic profiles of adjuvant Atezolizuma in combination with standard anthracycline/paclitaxel adjuvant chemotherapy *versus* chemotherapy alone in early-stage TNBC, which currently had a more positive treatment effects, but the primary endpoint, EFS, and other efficacy endpoints continue to be followed up (Mittendorf et al., 2020).

Pembrolizumab has received a considerable amount of attention from investigators since its introduction to the market A phase I study evaluated patients who received prior anthracycline therapy and had received ≤2 lines of prior chemotherapy for metastatic disease. After 6 weeks of Dox + Pembrolizumab combination therapy, it was found to be well tolerated and had a more positive treatment outcome in mTNBC patients who had not received anthracycline therapy, but longer follow-up studies are needed (Yuan et al., 2022). In addition, the KEYNOTE-173 trial (Schmid et al., 2020) combined Pembrolizumab with chemotherapy (12 cycles of paclitaxel + doxorubicin + cyclophosphamide) in patients with high-risk, early-stage TNBC, which demonstrated manageable toxicity and favorable anti-tumor activity. Meanwhile, the pCR rate was positively correlated with tumor PD-L1 expression and sTIL levels. KEYNOTE-522 is a multicenter, randomized, controlled Phase III clinical study (Schmid et al., 2022). It is the first to explore the use of Pembrolizumab in combination with chemotherapy (4 cycles of paclitaxel + carboplatin sequential doxorubicin + cyclophosphamide (TP) in patients with early-stage triple positive breast cancer, and the results of the trial showed a higher pCR rate in the pembrolizumab + chemotherapy group compared to the placebo + chemotherapy group (64.8% vs. 51.2%, EFS (84.5% vs. 76.8%). The similar study design and different results suggest that the use of immunotherapy in the neoadjuvant treatment of TNBC needs to be further explored and supported by long-term survival follow-up results.

Durvalumab could block the binding of PD-L1 to PD-1 and CD80, intercepting tumor immune escape and releasing suppressed immune responses. It has shown positive therapeutic effects in the treatment of TNBC in the clinic (Ghebeh et al., 2022). A Phase I/II trial evaluated the safety and efficacy of concomitant neoadjuvant treatment with Durvalumab in combination with paclitaxel and doxorubicin/cyclophosphamide (ddAC) in patients with stage I-III TNBC. The study found that no dose-limiting toxicities were observed in the Phase I trial. In Phase II, the neoadjuvant chemotherapy regimen group of durvalumab + chemotherapy had a pCR rate of 44%, and the pCR rate was higher in cancers with high sTIL (Foldi et al., 2022). GeparNuevo is a multicenter, prospective, randomized, double-blind, placebo-controlled Phase II trial (Pusztai et al., 2018; Loibl et al., 2019). Durvalumab (1.5 g, Q4W) was found in combination with albumin paclitaxel (125 mg/m2, QW) for 12 weeks, followed by 4 cycles of Durvalumab added to doxorubicin/cyclophosphamide for the treatment of cT1b-cT4a-d in patients with triple positive breast cancer. The 3-year iDFS was 95.5% for pCR patients in the group using Durvalumab in combination with basal chemotherapy and 86.1% for those not using it (HR 0.22, 95% CI 0.05-1.06). The neoadjuvant regimen of Durvalumab in combination with chemotherapy as far as possible significantly improved the survival of TNBC patients. However, more studies are needed in later stages to elucidate the optimal duration and sequence of Durvalumab in the treatment of patients with early TNBC (Ghebeh et al., 2018). Although the advantages and effects of Durvalumab in the treatment of TNBC have been observed in clinical trials, which is a new hope for the majority of patients, the effectiveness of Durvalumab needs to be confirmed by the results of many clinical trials in order to be successfully marketed. Progress in clinical trials of DOX liposomes in combination with PD-L1 inhibitors for the treatment of triple positive breast cancer was shown in Table 1.

**TABLE 1:** Progress of clinical trials of DOX liposomes in combination with PD-L1 inhibitors for the treatment of triple positive breast cancer.

PD-L1 inhibitor	Trial	Stage	Patient population N)	Intervention	Outcome measures
Atezolizumab	ALICE NCT03164993 Røssevold et al. (2022)	the randomized, double-blind phase 2b (n = 79)	Stage II or III TNBC	Pegylated liposomal Doxorubicin (PLD)/Cyclophosphamide + Atezolizumab (Atezo-chemo; n = 40) VS. placebo (placebo-chemo; n = 28)	After 15 months was 14.7% (5/34; 95% CI 6.4%–30.1%) in the Atezo-chemo arm *versus* 0% in the placebo-chemo arm
Atezolizumab	IMpassion030 NCT03498716 Saji et al. (2021)	A global, prospective, randomized, open-label, phase III (n = 2300)	Stage II or III TNBC	paclitaxel 80 mg Saji et al. (2021) ubicin 60 mg/m2+cyclophosphamide 600 mg/m2 for 4 doses (2 weeks)+Atezolizumab or placebo	The invasive disease-free survival (iDFS) and secondary endpoints include, iDFS in the PD-L1 selected tumour status (IC1/2/3) and node-positive subpopulations, overall survival, safety, patient functioning and health related quality of life (HRQoL). Progress of the trial is still being assessed
Atezolizumab	IMpassion031 NCT03197935 Mittendorf et al. (2020)	Randomized Phase III 1:1 (n = 333)	Stage II or III TNBC	Nabpaclitaxel + carboplatin + pembrolizumab-AC + pembrolizumab-surgery Atezolizumab plus standard anthracycline/taxane adjuvant chemotherapy *versus* chemotherapy alone in early stage TNBC	Median follow-up was 20.6 months (IQR 8.7-24.9) in the Atezolizumab plus chemotherapy group and 19.8 months (8.1-24.5) in the placebo plus chemotherapy group
Pembrolizumab	NCT02648477 Yuan et al. (2022)	Phase I/II (n = 10)	The patients with previously untreated stage II or stage III TNBC	Patients received Pembrolizumab 200 mg and 50–60 mg/m^2^ Dox every 3 weeks, followed by Pembrolizumab maintenance until progression or unacceptable toxicity	The combination of Pembrolizumab + Dox was well tolerated and had modest activity in anthracycline-naïve patients with mTNBC
Pembrolizumab	KEYNOTE-173 NCT02622074 Schmid et al. (2020)	Phase I/II (n = 60)	High-risk, early-stage, non-metastatic TNBC	Patients received Pembrolizumab 200 mg (cycle 1) and paclitaxel Schmid et al. (2020) orubicin and cyclophosphamide (12 weeks) + surgery	pembrolizumab + chemotherapy for high-risk, early-stage TNBC showed manageable toxicity and promising antitumor activity, and the pCR rate showed a positive correlation with tumor PD-L1 expression and sTIL levels
Pembrolizumab	KEYNOTE-522 NCT03036488 Schmid et al. (2022)	Phase I (n = 1,174)	TNBC patients wi`th T1c N1-2/T2 N0-2	the pembrolizumab-chemotherapy (TA) (n = 784) VS. Ngroupthe placebo-chemotherapy group (n = 390)	the percentage with a pathological complete response was significantly higher among those who received Pembrolizumab plus neoadjuvant chemotherapy than that of the control group
Durvalumab	MEDI4736 Foldi et al. (2022)	Phase I/II (n = 59)	Stage I to III TNBC	Durvalumab concurrent with weekly nab-paclitaxel and dose-dense doxorubicin/cyclophosphamide (ddAC) neoadjuvant therapy	Complete pathological remission (CPR)rate can be increased by 44% (95% CI: 30%–57%), Which is higher in cancers with high sTIL.
Durvalumab	GeparNuevo NCT02685059 Loibl et al. (2019)	Multicenter, prospective, randomized, double-blind,Phase II trial (n = 174)	TNBC patients with cT1b-cT4a-d	durvalumab 1.5 g Loibl et al. (2019) W plus nab-paclitaxel (125mg/m2, QW)for 12 weeks, followed by durvalumab/placebo plus epirubicin/cyclophosphamide followed by surgery	iDFS was 85.6% in the divalizumab group and 77.2% in the placebo group (HR 0.48, 95% CI 0.24-0.97, *p* = 0.036); DDFS was 91.7% vs. 78.4% (HR 0.31, 95% CI 0.13-0.74, *p* = 0.005)

## 4 Conclusion and outlook

Nano-delivery systems are a promising cancer therapy due to their versatility and physicochemical properties. As a research center for the treatment of TNBC, its anticancer potential is becoming increasingly prominent. In this thesis summarizes elaborated the progress of novel nanoparticles, liposomes, micelles, dendrimers polymers, nanogels, exosomes and other delivery systems based on the chemotherapeutic drug DOX in the treatment of TNBC. The research progress of clinical trials of Immune Checkpoint Inhibitor combined with DOX in the treatment of TNBC is also summarized. With a view to expanding the depth and breadth of DOX in the treatment of TNBC, laying a theoretical foundation for clinical trials.

Although great strides have been made in the treatment of TNBC by the DOX-based nano-drug delivery systems, there are still some bottlenecks and challenges in their preparation and translation into clinical applications. For example, 1) nanoparticles can be used to control the release rate and targeting of DOX, which can improve the accumulation of drugs in tumor tissue, further reduce the damage to normal tissues. The surface of the nanoparticles is modified in order to achieve various therapeutic strategies (targeted delivery, drug combination therapy or photothermal therapy). The application of DOX nanoparticals for the treatment of TNBC *in vivo* and *in vitro* has come a long way. However, the nanoparticles therapy translate from preclinical studies into clinical test, they are still need to conduct more complex experiments, which ensure its safety in tumor treatment. Includes assessment of species differences between laboratory animals and humans, individual differences between patients. Long-term prognostic conditions of the therapy for tumor patients, such as tumor recurrence, distant metastasis and cumulative toxicity. (2) Liposomes form a specific oil-in-water structure, which can well load hydrophilic and lipophilic drugs, increase bioavailability and targetability of these drugs. DOX in liposomes reduces its cardiotoxicity through accumulation in tumor tissues increase and distribution in normal tissue decrease. With the development of nanomedicine, nano-liposomes have shown significant improvement in encapsulation rate, stability, targeting, controlled drug release, and reduction of phagocytosis by the reticuloendothelial system, which has accelerated the application of DOX liposomes in the clinic. The efficacy of intravenous liposomes, however, is limited because it are still easily cleared by the reticuloendothelial system. Besides, free liposomes in the blood is unstable to broke assembly and cause drugs-release early. This problem limits the promotion and clinic application of DOX-liposomes in tumor treatment. 4) Nanogels have a larger surface area, broad three-dimensional network structure, good solubility and biocompatibility when compared with other nanoparticles. Therefore, nanogels can avoid the removal of DOX-chemical modificated by MPS, and realize its ring-controlled release, which enhances the effect of treating TNBC. Nevertheless, there are still barriers for nanogels from the experimental stage to clinical application. Firstly, due to the property that nanogels can be retained in the body for a long period of time, such a “slow and controlled release”capability may, to some extent, result in the accumulation of drug-carrying nanogels in some tissues and sites (such as liver and spleen). Secondly, although the short-term biocompatibility of nanogels can be assessed well in animal models, its long-term biocompatibility cannot be guaranteed in these models.5) Dendritic polymer drug delivery systems with large internal cavity structure and dense surface active functional groups are well suited for loading drugs in their internal cavities. In addition, their surface active functionalities can covalently bind/electrostatically complex with the drug, which can significantly improve drug solubility and stability. But compared to more mature drug delivery systems such as nanoparticles, liposomes and micelles, the development of dendrimers for drug delivery has been slow, and few dendrimer formulations are commercially available. The main reason may be the most dendrimers are nondegradable. Thus, as an anti-cancer drugs’s delivery system, its risk of accumulation in the body will greatly increased.6) Exosomes, as a type of extracellular vesicles, are characterized by high biocompatibility, low immunogenicity, and surface ligand specificity to deliver drugs into target cells. To facilitate the clinical translation of exosomes, how to select specific TNBC cell surface markers or receptors to enhance their targeting. In addition, there is a need to design suitable exosome surface modules to exclude potential side effects of exosomes. More importantly, the binding efficiency and specificity of TNBC cell surface biomarkers should be maximized without destroying the structure and contents of exosomes. These are the research focuses for researchers to construct novel exosome delivery systems.7) Compared with other nanomaterials, inorganic nanomaterials (graphene oxide (GO), carbon quantum dots (QDs), carbon nanotubes (CNTs), nanorods, etc.) are ideal materials for drug delivery systems. Because of their advantages such as simple preparation, superior stability, high drug loading capacity, good biocompatibility and easy surface modification. Although these inorganic nanomaterials are gradually finding applications in bioimaging and diagnostics for TNBC therapy. There are still many challenges in clinical translation for the nanomaterials, the biggest of which are its long-term potential toxicity and low clearance *in vivo*. Numerous studies have demonstrated the low acute toxicity of these inorganic nanomaterials, however, whether it can be cleared from the body and whether it causes long-term toxicity cannot be verified. Therefore, when the clearance rate of inorganic nanomaterials are evaluated clearly, it is applied for clinical studies will be possible.

In summaries, DOX-based nano drug delivery system can effectively inhibit tumor proliferation and metastasis, inhibit the growth, metastasis and recurrence of drug-resistant clonal tumor cells through multiple pathways and mechanisms. Among them, there maybe have four directions for future research and development. Firstly, the targeting of nano-delivery system can be enhanced to confers it the ability to target tumor tissues, and reduces the toxicity of DOX on normal tissues and cells, especially the heart. Secondly, when DOX, the basic chemotherapeutic agent for the treatment of TNBC, is used in combination with photothermal therapy, it can inhibit and kill TNBC cells in a variety of ways, especially by removing tumor stem cells. In this condition, the developments of DOX-chemical therapy are expected to mitigate or tackle tumor proliferation, metastasis and recurrence. In addition, nano-delivery systems enabling immunotherapy in combination with chemotherapy have become a new trend, especially for PD-1/PD-L1 in the treatment of TNBC, as PFS OR, pCR and other indicators have previously shown better efficacy, but chemotherapy in combination with immunotherapy has some challenges. For example, we have to understand which population is the real need for immunotherapy patients and, more importantly, what is the optimal solution in terms of the order of administration of chemotherapy and immunotherapy. Therefore, more clinical trials are needed for validation. At the last, combining gene therapy with DOX chemotherapy can significantly improve the precision and intelligence of TNBC treatment. With the development of biomedicine, delivery systems such as nanoparticles, liposomes, micelles, gels, dendritic polymers and exosomes will be further optimized in terms of improving drug targeting, solubility, stability and bioavailability. By deeply studying the characteristics of various delivery systems and applying them, it will provide more possibilities for the treatment of TNBC.
