# Corrigendum: Advances in Doxorubicin-based nano-drug delivery system in triple negative breast cancer

**DOI:** 10.3389/fbioe.2025.1623425

**Published:** 2025-06-02

**Authors:** Weiwei Zeng, Yuning Luo, Dali Gan, Yaofeng Zhang, Huan Deng, Guohui Liu

**Affiliations:** ^1^ Department of Pharmacy, Shenzhen Longgang Second People’s Hospital, Shenzhen, Guangdong, China; ^2^ Shenzhen Longhua Maternity and Child Healthcare Hospital, Shenzhen, Guangdong, China

**Keywords:** doxorubicin, nanocarriers, triple negative breast cancer, drug delivery, clinical

In the published article, there were two errors.

A correction has been made to **Abstract**.

This sentence previously stated:

“Triple positive breast cancer (TPBC) is one of the most aggressive breast cancer.”

The corrected sentence appears below:

“Triple negative breast cancer (TNBC) is one of the most aggressive breast cancer.”

A correction has been made to **Introduction**, Paragraph one.

This sentence previously stated:

“Triple positive breast cancer (TPBC) is a specific type of breast cancer that lacks estrogen receptor (ER), progesterone receptor (PR), and human epidermal growth factor receptor-2 (HER2), and accounts for approximately 15% of all breast cancer cases.”

The corrected sentence appears below:

“Triple negative breast cancer (TNBC) is a specific type of breast cancer that lacks estrogen receptor (ER), progesterone receptor (PR), and human epidermal growth factor receptor-2 (HER2), and accounts for approximately 15% of all breast cancer cases.”

The authors apologize for this error and state that this does not change the scientific conclusions of the article in any way. The original article has been updated.

